# Conditional versus non-conditional incentives to maximise return of participant completed questionnaires in clinical trials: a cluster randomised study within a trial

**DOI:** 10.1186/s13063-023-07604-6

**Published:** 2023-11-07

**Authors:** Johanna Cook, Jonathan A. Cook, Emily Bongard, Carl Heneghan, Chris C. Butler

**Affiliations:** 1 The Nuffield Department of Primary Care Health Sciences, University of Oxford, Radcliffe Observatory Quarter, Woodstock Road, Oxford, OX2 6GG UK; 2 Centre for Statistics in Medicine, Nuffield Department of Orthopaedics, Rheumatology and Musculoskeletal Sciences, University of Oxford, Oxford, UK

**Keywords:** Incentives, SWAT, Conditional incentive, Non-conditional incentive, Participant completed questionnaire

## Abstract

**Background:**

High participant retention enhances the validity of clinical trials. A monetary incentive can increase retention, but it is not known if when it is provided and if it is conditional matters. We aimed to determine whether there was a difference in the number of follow-up trial questionnaires returned when a monetary (gift voucher) incentive was given to participants at recruitment (non-conditional), compared to informing participants at recruitment that the incentive would be given only once their 14-day daily diary (questionnaire) had been returned (conditional).

**Method:**

A cluster randomised study within a trial embedded within the Antivirals for influenza-Like Illness, An rCt of Clinical and Cost effectiveness in primary CarE (ALIC^4^E) Trial. Matched site pairs (GP practices) were randomised using computer-generated random numbers, to either a non-conditional or conditional monetary voucher incentive (only once their 14-day daily diary (questionnaire) had been returned. Sites were matched on previous recruitment levels and practice list size. Analyses were conducted according to randomised groups irrespective of compliance with a two-sided 5% level statistical significance level. The main analysis of the primary outcome (site proportion of diaries returned) was linear regression accounting for site pair (using cluster-robust variance). Additional weighted, paired and non-parametric sensitivity analyses were conducted. Secondary outcomes were the site average number of completed pages, time to return diary, and cost related to the incentive (administration and postage).

**Results:**

Of the 42 randomised sites (21 for each intervention), only 28 recruited at least one participant with only 10 practice pairs recruiting participants at both constituent sites. Raw diaries return proportions were 0.58 (127/220) and 0.73 (91/125) for non-conditional and conditional incentive groups. Regression analysis adjusted for site pair showed no significant difference in returns, − 0.09, (95% CI, − 0.29, 0.10, *p* = 0.34); when weighted, there was still no clear difference: 0.15 (95% CI, − 0.02, 0.31, *p* = 0.07). There was no clear statistical evidence of a difference in time taken to return questionnaires, nor the proportion of pages completed, by the intervention group in the main analyses (all *p* > 0.05). The conditional incentive was approximately £23 cheaper per diary returned based upon observed data.

**Conclusion:**

There was no clear evidence of a statistically significant difference in the proportion of participant-completed diaries returned between conditional or non-conditional incentive groups. The time to questionnaire return and completeness of the returned questionnaires were similar in both groups. There was substantial statistical uncertainty in the findings. Some of the sensitivity analyses suggested that a meaningful benefit of a conditional incentive of a magnitude that would be meaningful was plausible. The conditional approach costs less in cash terms.

**Supplementary Information:**

The online version contains supplementary material available at 10.1186/s13063-023-07604-6.

## Background

Completeness of follow-up data is vital to trial validity. Despite this, most trials have non-trivial levels of missing data (called ‘loss to follow-up’ or ‘attrition’) [1]. Higher levels of missing data increase the risk that the observed results are unrepresentative of the whole study group [2]. There are many reasons why data might be incomplete at the end of a trial; from trial sites not collecting all the data, to equipment failure, to data going missing or degrading during the time of the trial, and also participants not responding to questionnaires. The way that data is lost within a trial has differential effects on the potential bias assumed from that missing data with data ‘missing not at random’ the most challenging to deal with [3]. When participants themselves do not respond to questionnaires this should be considered data that is ‘missing not at random’ because there could be reasons for disproportionate levels of response from the different groups and, as a result, an increased likelihood of bias is assumed within the trial results.

There are many different methods that have been suggested or are used to improve retention rates in clinical trials, especially where the follow-up is through participant-completed questionnaires, but reports of the effectiveness of many of these are anecdotal [4]. In their Cochrane review, Brueton et al. [5] examined the methods for improving loss to follow-up that have been evaluated in randomised studies or quasi-randomised studies in health care. In the studies reviewed retention was listed as either a primary or secondary outcome. Of the trials identified, 14 evaluated incentives (monetary and non-monetary) that aimed to increase the response rate to participant-completed postal questionnaires. They concluded that of all strategies investigated for the return of questionnaires, the addition of a monetary incentive increased responses the most. This confirms findings from the previous Cochrane Review by Edwards et al. [6] that found that the return of postal questionnaires increased (potentially more than doubling the odds of return) when a monetary incentive was provided. A conclusion of the Brueton et al. [5] review was that more research was needed into methods for using incentives, and they expressed the need for more research into the optimal time monetary incentives should be provided, given the potential cost difference involved between providing an incentive initially for all, or just providing an incentive as a reward only for returned questionnaires. Trials currently being implemented in primary care vary in providing conditional [7, 8] or non-conditional [9] incentives. Trial teams often debate which approach is optimal.

We therefore set out to examine the effect of adding in a monetary incentive of £20 as either conditional (given only once their questionnaire is returned) or non-conditional (given to all participants at the point of recruitment) as a sub-study nested within a large international randomised controlled trial, the Antivirals for influenza-Like Illness? An rCt of Clinical and Cost effectiveness in primary CarE (ALIC^4^E) Trial (ISRCTN:27908921) [10, 11]. The incentive was given in the form of a monetary voucher that could be redeemed at many different high street shops. Specifically, we aimed to determine the proportion of symptom diaries returned, the time to receipt of the diaries, the effect on pages completed, and cost differences between conditional and non-conditional approaches, including voucher costs and administration time.

## Method

### Basic design

This was a cluster-randomised matched-pair parallel-group Study Within A Trial (SWAT) [12, 13] within the ALIC^4^E Trial [10, 11]. SWATs use the sample provided by the host trial to study the approach to providing a monetary incentive (Tables 1 and 2). As a result, they do not typically have their own sample size or power calculation and proceed on an opportunistic basis as was the approach in this study. Our study proceeded according to a pre-specified protocol [13], that was approved by the research ethics committee as part of the main trial protocol, and which did not change during the course of the study.

**Table 1:** Interventions

**Non-conditional incentive arm**	A £20 gift voucher given to study participants at the end of the recruitment visit (Baseline)
**Conditional incentive arm**	Participants informed that a £20 gift voucher would be given upon the return of the trial symptom diary (Diary Return), and voucher then sent to those who returned a diary by the trial team

**Table 2:** Objectives and outcome measures

**Primary objective:** To assess the effect on study questionnaire return of giving a participant in an influenza trial a £20 monetary incentive at the recruitment visit (non-conditional) or only once they have returned their trial diary (conditional)	**Primary outcome measure:** Proportion of participants returning a symptom diary
**Secondary objective:** To assess the effect of an incentive on: 1. The time taken to return the participant diary 2. The proportion of the diary content completed	**Secondary outcome measure:** 1. Time to diary return (days) 2. The completeness of the returned diaries (number of pages completed, total possible: 36 pages)
**Exploratory objective:** To investigate the cost difference between conditional and non-conditional incentives	**Exploratory outcome measures:** Costs incurred as a result of the incentives given

ALIC^4^E was a European multi-national, multi-centre, phase IV, open-labelled, pragmatic, adaptive-platform, individually randomised RCT. The trial aimed to determine whether adding antiviral treatment to the best usual primary care is clinically and cost-effective for patients with influenza-like illness. The trial was implemented in 21 networks across 15 countries in Europe. Networks were organisations, such as a university or health centre, that managed the recruitment of participants locally into the main trial. The follow-up requirement within the ALIC^4^E Trial was a 14-day paper symptom diary (questionnaire) completed by the participants or their parent or legal guardian for the 2 weeks following recruitment into the study, then two further questionnaires completed via telephone call at days 14 and 28. The symptom diary collected both primary and secondary endpoint data. See Table 6 in Appendix 1 for ALIC^4^E inclusion and exclusion criteria and Table 7 in Appendix 2 for the full list of trial procedures.

We implemented the SWAT only in the UK networks that participated in the ALIC^4^E Trial. Though collectively the recruiting networks achieved a return rate of the symptom diaries of over 90% in the first winter season of recruitment, within the UK sites the return rate was 69%, meaning a loss to follow-up rate of 31%, far higher than the standardly accepted level of 20% or less [14]. This was despite the same pre-planned follow-up procedure for all networks. Despite discussion between the recruiting networks in the study, we were unable to confirm a reason for the lower follow-up rate in the UK network. For the SWAT we indicated in the UK in participant information sheets that vouchers would be given to participants to thank them for their participation within both arms. We also indicated in the reminder texts for the participants in the conditional incentive group that once they returned their diary, they would receive their voucher. Generic reminder text messages were sent to all participants recruited in the UK on days 1, 9 and 21. The recruiters were asked to complete receipts for all the vouchers dispensed so that the trial team could monitor compliance from the trial sites with voucher issue.

The main challenges in the design and implementation of this SWAT were ensuring that the individual participants received the vouchers at the appropriate time and that the randomisation arms had roughly equal levels of recruitment of participants into them. A cluster randomised design was used to enable the intervention to be applied consistently within sites for practical reasons. As there was concern about the relatively low number of clusters, and in order to try to manage anticipated differences between sites, which might impact upon recruitment, a matched pair cluster randomised design was used.

All outcomes were assessed after participants had completed their trial involvement. There were no changes to these outcomes after the SWAT commenced. Additional file 1 includes the CONSORT checklist for reporting.

### Recruitment

The ALIC^4^E UK network included GP surgeries in Oxford, Southampton and Cardiff networks. The SWAT operated in 42 out of the 43 GP Surgeries which were recruited into the ALIC^4^E Trial in the UK during the second and third seasons of the trial. One was excluded because of the non-availability of a further practice necessary for a matched pair. The site selected for exclusion was the last one recruited into the trial in Season 3. Recruitment of participants was during periods of heightened influenza activity, but all sites were randomised and trained prior to the participant recruitment beginning in season 2. Recruitment finished at the end of the third influenza season in 2018 and once the host trial had reached its recruitment target. Participants taking part in this SWAT had no additional requirements to those detailed above.

### Outcomes

The primary outcome of interest was the proportion of diaries returned by a site. Secondary outcomes were the site average number of completed pages, time to return diary, and cost related to the incentive. The cost was calculated by estimating staff administration time and postage.

### Randomisation

This SWAT used a matched pair cluster randomised parallel group design. The trial sites were the unit of randomisation. The sites were cluster-randomised within matched pairs in order to keep the process as straightforward as possible for the recruiters. Randomisation was performed in two waves (before the start of seasons 2 and 3) using computer-generated random numbers carried by one of the investigators (JAC). We paired the recruiting sites from Season 1 according to previous levels of recruitment and they were then randomised within these pairs prior the start of season 2. Sites were paired with a site with the next nearest level of previous recruitment. New sites starting during seasons 2 and 3 were randomised in matched pairs, but using the practice list size as the indicator of potential recruitment. Randomisation took place before the beginning of the second recruitment season. Sites continuing from season two carried on in their original randomised group. If there were to be an uneven number of sites and so an unpaired site, this site would continue on in the trial with the incentive given at a time more convenient for them, but they would not be included in the analysis of this SWAT. GP practices were not blinded to their allocation due to the necessity of them either distributing the incentives initially or not. Participants were unaware that there was a SWAT taking place as it was thought this might influence whether or not they returned their diary.

### Statistical analysis

All statistical analyses were based upon the randomised groups irrespective of compliance with allocation at the cluster (site) level. They were carried out using Stata version 15. Overall study and intervention group level data were summarised using a number of events and percentage for binary variables, mean and standard deviations (SDs) or median and interquartile range (IQR) or ranges at site and participant level where appropriate.

The primary outcome was analysed as the proportion of returned diaries at the site level. Secondary outcomes were average number of completed pages, average time to diary return (days) and the average total cost (£) also at the site level. For all secondary outcomes, analyses were based also upon sites where at least one diary was returned. Whether the symptom diary was returned, and if it was, when it was received and how many pages were completed was available for all participants. Diaries were due to be returned at the earliest on day 15 after randomisation. A completed page was one where all possible data had been entered by the participant. Where a participant had no more symptoms they could indicate this at the top of the page and not answer any more questions, this would also be considered a complete page. There was no imputation of outcome data for sites where no one was recruited, or where a participant did not return their symptom diary.

Due to the unanticipated small number of available pairs with both sites with outcome data, the main analysis of the primary and secondary outcomes was carried out using linear regression to estimate the mean difference in the proportion between the intervention groups. The intervention group was the sole explanatory variable. The linear regression was carried out in Stata using the cluster option to implement cluster robust variance estimation to account for site pair (a paired comparison had originally been planned). A sensitivity analysis for the primary and secondary outcomes used the same regression model but weighted the observations according to the number recruited or number of diaries returned depending upon the outcome. Other sensitivity analyses were also carried out for all the outcomes. These were a Mann–Whitney *U* test on the site level data (ignoring the pairing) and two analyses on the subset of available paired data (paired *t* test and a Wilcoxon signed rank test). Ninety-five per cent confidence intervals (CIs) were calculated from the regression and the paired t-test analyses for the mean difference. Statistical significance was assessed at the 2-sided 5% significance level throughout. Recruitment data was available for all sites.

### Cost analysis

When assessing the cost of each intervention, a full economic analysis was not undertaken. We assessed the gross cost impact per diary in a simple cost analysis. When considering this there were different factors to take into account. The first and most important was the cost of the vouchers themselves, £20 per voucher dispensed. The indirect costs must also be taken into consideration, administration time and the cost of postage.

In the non-conditional arm, it was the recruiter, most commonly a GP, who was giving out and completing the required forms and receipts for each voucher given. Five minutes would be allowed for an administrative task such as this when considering NHS service support cost reimbursements. At this time, a GP time was reimbursed at £80 per hour according to the NHS research reimbursement figures.

In the conditional arm, the amount of time required for the administration was a similar 5 min, but this task would be completed by an administrator whose time was charged at £21 per hour. In addition to this, there was the postal cost of sending the voucher to the participants for which the cost of a second class letter was £0.56.

To assess the impact of the direct and indirect costs, we took into account the number of times each item or activity was required, according to the number of vouchers given and so calculated a total cost per arm and then a total cost per questionnaire returned. No formal analysis of the results was carried out and the cost per diary returned for each group was calculated.

## Results

**Fig. 1:**
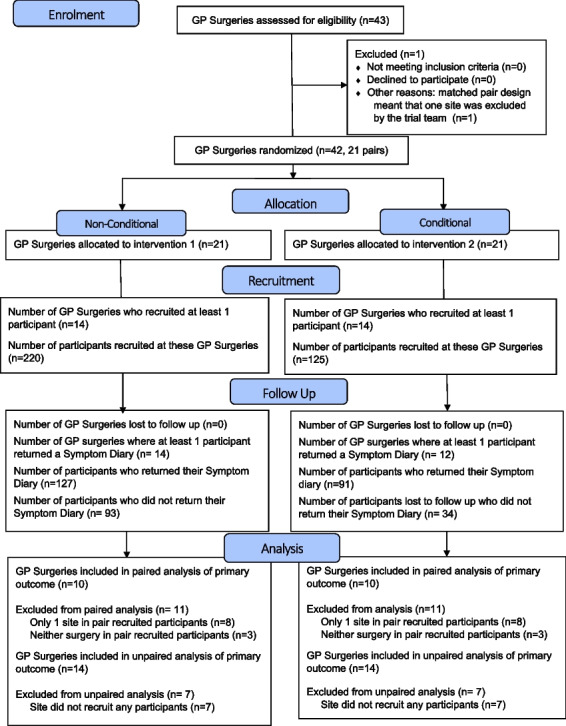
Consort flow diagram

The Consort Diagram is provided in Fig. 1 in Appendix 3. The baseline characteristics of previous recruitment were relatively well balanced though practice size was not with the conditional incentive group having a higher medium size (Table 3). The baseline characteristics of the participants within the SWAT were well reasonably well balanced though there is some difference in the median age of the participants (Table 4). Overall 335 participants were recruited from 28 sites; 220 and 125 in the non-conditional and conditional monetary incentives arms, respectively. The baseline participant-level data was reasonably well balanced between intervention groups.

**Table 3:** Baseline characteristics of sites

		Non-conditional incentive arm (*n* = 21)	Conditional incentive arm (*n* = 21)
Previous recruitment — median (range) (for sites that recruited in season 1)	All	3 (1–19), *n* = 9	3 (1–14), *n* = 9
	Only those where both sites in cluster pair recruited	7 (1–19), *n* = 5	7 (2–14), *n* = 5
Practice list size — median (IQR)	All	9674 (7900, 15,408), *n* = 21	12,055 (7050, 15,600), *n* = 21
	Only those where both sites in cluster pair recruited	11,350 (8751, 16,077), *n* = 10	12,528 (8862, 16,341), *n* = 10

*IQR* interquartile range

**Table 4:** Baseline characteristics of participants^a^

	Non-conditional incentive arm (*n* = 220 unless otherwise stated)	Conditional incentive arm (*n* = 125 unless otherwise stated)
Duration of illness (days) — median (IQR)	2 (2, 3)	3 (2, 3)
Score of clinical severity — median (IQR)^b^	2 (1, 2)	2 (2, 2)
Age (years) — median (IQR)	29 (12, 46)	36 (23, 53)
Temperature — median (IQR)	37.1 (36.6, 37.6)	37.6 (37.0, 38.2), *n* = 124
Female — *n* (%)	135 (61)	73 (58), *n* = 124

^a^ If returned; *IQR* interquartile range

^b^ score = no (1), minor (2), moderate (3) or major (4) symptoms

Of the 42 sites involved in the study, only twenty-eight (67%) sites recruited at least one participant (Table 5). Only 10 of the 21 (48%) site pairs had at least one participant recruited in both sites within the pair. The median (IQR) numbers recruited across all sites was 2 (0, 10), and by conditional and non-conditional arms were 2 (0,9) and 3 (0, 10), respectively.

**Table 5:** Overall recruitment and outcomes by intervention group at the participant level

	Participants recruited across sites	Diaries received across sites — *n* (%)	Days to diary return — median (IQR)^a^	Number of pages completed — median (IQR)^a^
Non-conditional incentive	220	127 (58)	23 (19, 27)	35 (35, 35)
Conditional incentive	125	91 (73)	20 (18, 25)	35 (35, 35)
Total	345	218 (63)	21 (18, 27)	35 (35, 35)

*IQR* interquartile range

^a^If returned

### Compliance

Compliance was defined as the site or trial staff giving or sending the participants their trial voucher at the time they were randomised to receive it. All participants in the conditional incentive group received their vouchers. Within the non-conditional group, there were 5 participants who did not receive the vouchers as they should have done. This was due to site non-compliance and non-response to the trial team who were trying to restock their vouchers. The site was re-trained and the issue did not occur again.

### Return of diaries

Twenty-six of the twenty-eight (93%) sites that recruited at least one participant returned one or more diaries; the proportion returned varied from 0 to 1.0 within both intervention groups reflecting the small number recruited per site (Table 5). Median and IQR site proportions returned were 0.66 (0.57, 1.00) and 0.71 (0.50, 0.80) for the non-conditional and conditional incentive groups, respectively. The raw proportion returned irrespective of site was 0.58 (127/220) and 0.73 (91/125) for non-conditional and conditional incentive groups. Corresponding values for the subset (10 pairs) where both of the constituent sites paired at randomised recruited one or more participants were 0.56 and 0.73. The median and IQR for the aforementioned subset of 10 pairs were 0.64 (0.57, 1.00) and 0.71 (0.50, 0.92) for the non-conditional and conditional incentive groups, respectively.

Linear regression analysis adjusted for cluster pair (unweighted) gave a mean difference in the proportion of diaries returned between interventions of − 0.09, 95% CI (− 0.29, 0.10), *p* = 0.34. The sensitivity analysis which was weighted by the number of individuals recruited at each site produced a substantially different raw effect though it was still not statistically significant: 0.15, 95% CI (− 0.02, 0.31), *p* = 0.07. Non-parametric test Wilcoxon signed rank test gave a similar finding of no clear statistical evidence of a difference (*p* = 0.76). The other sensitivity analyses which ignored the pairs and analysed all sites where one or more participant was recruited also gave similar findings with Mann–Whitney *p* = 0.69, and unweighted paired *t* test had a mean difference of − 0.04, 95% CI (− 0.28, 0.19), *p* = 0.70.

### Time to diary return

The individual time to return ranged from 10 to 64 days across both intervention groups. Median and IQR of the average times to return by intervention group at site level were 23 (20, 26) days and 21 (19, 25) days for the non-conditional and conditional incentive groups, respectively. Corresponding values for the subset (9 pairs) where both constituent sites had at least one diary returned were 24 (23, 25) days and 21 (19, 24) days for the non-conditional and conditional incentive groups, respectively.

Regression analysis adjusted for site pair was a mean difference between interventions of − 0.9, 95% CI (− 5.7, 3.8) days, *p* = 0.69. The sensitivity analysis which was also weighted by the number of individuals recruited at each site produced a substantially different raw effect which was statistically significant and favoured the conditional incentive group: − 3.1, 95% CI (− 5.0, − 1.1), *p* = 0.004. Non-parametric test Wilcoxon signed rank test gave a similar finding as the main analysis (*p* = 0.21). The other sensitivity analyses which ignored the pairs and analysed all sites where one or more participant was recruited also gave similar findings with Mann–Whitney *p* = 0.35, and unweighted paired *t* test had a mean difference of − 0.8, 95% CI (− 7.0, 5.4) days; *p* = 0.77.

### Number of pages completed

The average number of pages completed per diary returned varied from 7 to 36 pages across both intervention groups. Median and IQR average pages completed by the intervention group at the site level were 35 (34, 35) pages and 35 (35, 35) pages for the non-conditional and conditional incentive groups, respectively. Corresponding values for the subset (9 pairs) where both constituent sites had at least one diary returned were 35 (34, 35) pages and 35 (35, 35) pages for the non-conditional and conditional incentive groups, respectively. When returned, the diary was generally fully completed or almost fully complete; only 7 (3%) across all participants failed to return at least 30 pages when the diary was returned. By intervention group, this was 4 (3%) and 3 (3%), respectively, for the non-conditional and conditional intervention groups.

Regression analysis adjusted for cluster pair was a mean difference between interventions of 0.5, 95% CI (− 1.2, 2.3) pages, *p* = 0.52. The sensitivity analysis which was also weighted by the number of diaries returned at each site had a similar result: 0.3, 95% CI (− 0.7, 1.2), *p* = 0.55. Non-parametric test Wilcoxon signed rank test gave a similar finding (*p* = 0.15). The other sensitivity analyses which ignored the pairs and analysed all sites where one or more participant was recruited also gave similar findings with Mann–Whitney *p* = 0.18, and unweighted paired *t* test had a mean difference of 0.2, 95% CI (− 0.7, 1.1) pages; *p* = 0.61.

### Cost analysis

In the non-conditional incentive arm, there were 220 participants recruited, of these 215 received a £20 voucher despite only receiving 127 diaries back. Therefore the total cost for the vouchers was £4300 and for each questionnaire actually returned the cost was £33.85. In the conditional incentive arm, because vouchers were only sent out once a diary was received, the total cost for the vouchers was £1820 and the cost for each diary received is £20.00.

For the indirect costs in the non-conditional arm with 215 vouchers distributed, the total cost for the administration time was £1433 and £11 per diary received. For the conditional arm, the total cost of the administration was roughly £159 and £1.75 per diary received. The total additional cost of the second class letter was £50.96 making a total of roughly £2.31 per diary received when considering administration and postage.

Therefore, the total cost for the non-conditional incentive arm was £5733 and per diary received was estimated to be £45. For the conditional incentive arm, the total was estimated to be around £2029 with a per diary cost of £22 a total difference of £23 in favour of the conditional arm.

## Discussion

### Impact on current literature

This was the first study to compare the effect of conditional versus non-conditional monetary incentives on return and completion of a participant-completed questionnaire in a RCT. There was no statistically significant difference in the proportion of questionnaires returned between the times at which the incentive is given in line with the current, limited, research. However, the raw difference of a 15% improvement (17% for the subset of site pairs where both recruited one or more) diaries returned in favour of the conditional incentive arm would be a meaningful one to trialists if genuine. This along with the confidence intervals for the site-level analyses being relatively wide suggests that the analysis lacks precision and that the possibility of a genuine and operationally important difference (to trialists) cannot be ruled out. Sensitivity analyses which are weighted by the number of observations that contributed to the site summary suggest that such an effect is quite possible. This needs exploring in further studies and incorporating this data ultimately within an updated systematic review of incentive use for retention. Additionally, the cost difference between the two approaches suggests that a conditional incentive is more economical.

### Strengths

This SWAT was implemented into the ALIC^4^E UK networks with very little to no impact on the trial, the study team and the recruiting sites and with none on the participants themselves. Compliance was very high which to a degree probably reflected the use of a cluster randomised design which made this much easier to administer, particularly part way through the study.

### Limitations

The sample size was relatively small, with only 345 participants recruited during the SWAT, and therefore, the study risks type II error. This is a general design challenge for SWATs, where typically no study-specific calculation is done for the sub-study itself and the recruitment is dependent upon the host trial.

Using the GP surgeries as the unit of randomisation was best for the practical implementation of the study, especially with consideration to the host trial and not wanting to compromise the main aims of that investigation. It was also an attractive method to use to overcome the known risk of imbalance due to differences between sites, but this, in turn, created its own difficulty. The way that the pairing of these sites worked out meant that there were many pairs with a site or both sites having not been recruited. This led to only being able to use results from only 10 of the 21 pairs recruited within the initial planned analysis. With the benefit of hindsight, the matched pair design was not the optimal choice due to the substantial number of sites that failed to recruit any participants and therefore did not contribute data to the analysis. In retrospect, this design in a primary care setting, where there is normally a relatively high number of sites that do not recruit *any* participants, was not well suited to the context. The limitations of the design in another context (community trials) have been noted [15]. It could work within secondary care, where most sites do recruit at least one or two participants. The paired design was chosen given the limited number of sites available but a potentially different design could have been to do either simple randomisation of all included sites or a stratified randomisation approach based again upon recruitment level; either may well have worked better. Allocation of sites to intervention by minimisation might also have achieved better balance at the site level. It is interesting to note that previous recruitment does not seem to have been a very strong predictor of future recruitment at least at the level of being useful for matching.

Analysing data from matched paired cluster trials is surprisingly difficult as Donner et al. show [16]. To simplify the statistical analysis, and without likely loss of statistical precision given the relatively small number of clusters and observations within clusters, all analyses were conducted at the cluster (primary care surgery) level. The sensitivity analyses using regression with cluster robust variance and weighting for the number of contributing observations may have been the most appropriate analysis in this content given the difficulties with the matching and low recruitment per site. A participant-level analysis would have been another option which was not utilised here.

The level of compliance with voucher issues was very high within this study, though not 100%. There were five participants within the non-conditional group who did not receive their voucher; none of these participants returned their diary. This lack of compliance could therefore have impacted upon the result. However, the analysis was carried out with these five participants included on an intention to treat as is generally recommended. Given the small number affected it seems unlikely to have affected the findings. No imputation for missing data was made which was a consequence of the design in terms of some sites not recruiting anyone.

## Conclusion

There was no clear evidence of a statistically significant difference in retention rate according to when a participant is given a monetary incentive for participant completed questionnaire return; however, our study may have lacked precision given the small number of clusters and that a substantial proportion did not recruit any participants. Additionally, there was no clear statistical evidence of a difference in the completion of the diary or questionnaire and the speed with which they were returned to the trial team. However, the analysis lacked precision, and sensitivity analyses which accounted for imbalances between sites suggested genuine effects are plausible. The possibility of a genuine and operationally important difference (to trialists) cannot be ruled out. Furthermore, there was, as expected, a large difference between the gross costs of giving a monetary voucher as a non-conditional incentive as opposed to a conditional incentive in simple monetary terms. It costs approximately £23 more for every diary received to give the vouchers as a non-conditional incentive. It may be cost-effective to give the incentives at the point at which a diary or questionnaire is received by the trial staff than when the participant is recruited. Further studies are needed to explore this aspect of clinical trial conduct.

### Supplementary Information

**Additional file 1: Table S1.** CONSORT 2010 checklist of information to include when reporting a cluster randomised trial.

## Data Availability

The datasets used and/or analysed during the current study are available from the corresponding author on reasonable request.

## Appendix 1

**Table 6:** ALIC^4^E Trial inclusion and exclusion criteria

Inclusion	Exclusion
• Male or Female, aged at least one year • Presenting with ILI* in primary care during a period of increased influenza activity * ILI = sudden onset of self-reported fever, with at least one respiratory symptom (cough, sore throat, running or congested nose) and one systemic symptom (headache, muscle ache, sweats or chills or tiredness), symptom duration of 72 h or less • Is able and willing to comply with all trial requirements • Participant or legal guardian(s) of a child is willing and able to give informed consent • Agrees not to take antiviral agents apart from study antiviral agents according to patient randomisation	• Chronic renal failure, e.g. known or estimated creatinine glomerular filtration rate < 60 ml/min (known = recorded in participant’s clinical records) • Condition or treatment associated with significant impaired immunity (e.g. long-term oral steroids, chemotherapy, or immune disorder) (known = recorded in participant’s clinical records) • Those who in the opinion of the responsible clinician should be prescribed immediate antiviral treatment • Allergic to oseltamivir or any other trial medication • Scheduled elective surgery or other procedures requiring general anaesthesia during the subsequent 2 weeks • Participant with life expectancy estimate by a clinician to be less than 6 months • Patient with severe hepatic impairment • Responsible clinician considers urgent hospital admission is required • Any other significant disease or disorder which, in the opinion of the responsible clinician, may either put the participants at risk because of participation in the trial, or may influence the result of the trial, or may affect the participant’s ability to participate in the trial • Involvement, including completion of any follow-up procedures, in another clinical trial of an investigational medicinal product in the last 90 days • Previous ALIC^4^E trial participation • Patients unable to be randomised within 72 h after onset of symptoms • Requirement for any live viral vaccine in the next 7 days • *Optional according to specific country legislation:* ◦Pregnant, lactating or breastfeeding women

## Appendix 2

### ALIC4E Trial procedures

**Table 7:** ALIC^4^E schedule of procedures according to the SPIRIT checklist

	Screening	Baseline (day 1)	Day 1–14	Day 14–28	Post day 28
Eligibility assessment^1^	✓				
Informed consent ^1+2^		✓			
Baseline CRF^1^		✓			
Physical examination^1^		✓			
Swab(s)^1^		✓			
Randomisation^1^		✓			
Dispensing of trial drugs^1^		✓			
Symptom diary^2^			✓		
Day 2–4 phone call^3^			✓		
Day 14–28 phone call^3^				✓	
After day 28 phone call^3^	✓				✓
Clinical notes review*^3^					✓
Adverse event assessments^3^			✓		✓
SAE follow-up^3^			✓		✓

^*^Country dependent

1 = Completed by recruiter

2 = Completed by participant, includes standardised written health-related quality of life assessment and documents resource use

3 = Completed by trial team (CI/PI/coordinator). Day 28 call includes standardised verbal health-related quality of life assessment

## Abbreviations

ALIC4E: Antivirals for influenza-Like Illness? An rCt of Clinical and Cost effectiveness in primary CarE
IQR: Interquartile range
RCT: Randomised controlled trial
SWAT: Study within a trial
